# Sol–Gel Synthesis of Endodontic Cements: Post-Synthesis Treatment to Improve Setting Performance and Bioactivity

**DOI:** 10.3390/ma15176051

**Published:** 2022-09-01

**Authors:** Xiaozhe Song, Aránzazu Díaz-Cuenca

**Affiliations:** 1 Materials Science Institute of Seville (ICMS), Joint CSIC-University of Seville Center, 41092 Seville, Spain; 2 Networking Research Center on Bioengineering, Biomaterials and Nanomedicine (CIBER-BBN), 28029 Madrid, Spain

**Keywords:** sol–gel synthesis, bioactive endodontic cement, calcium silicates, SBF, bioceramics

## Abstract

The sol–gel process is a wet chemical technique that allows very fine control of the composition, microstructure, and final textural properties of materials, and has great potential for the synthesis of endodontic cements with improved properties. In this work, the influence of different sol–gel synthesis variables on the preparation of endodontic cement based on calcium silicate with Ca/Si stoichiometry equal to 3 was studied. Starting from the most optimal hydraulic composition selected, a novel second post-synthesis treatment using ethanol was essayed. The effects of the tested variables were analyzed by X-ray diffraction, infrared spectroscopy, scanning electron microscopy, nitrogen physisorption, and Gillmore needles to determine the setting time and simulated body fluid (SBF) immersion to measure the bioactive response in vitro. The results indicated that the sol–gel technique is effective in obtaining bioactive endodontic cements (BECs) with high content of the hydraulic compound tricalcium silicate (C3S) in its triclinic polymorph. The implementation of a novel post-synthesis treatment at room temperature using ethanol allows obtaining a final BEC product with a finer particle size and a higher CaCO_3_ content, which results in an improved material in terms of setting time and bioactive response.

## 1. Introduction

Bioactive endodontic cements (BECs) are dental materials widely used in endodontics for vital pulp therapy, coronal sealing during regenerative endodontic procedures isolating the intracanal treatment from the coronal restoration, apical barriers in immature teeth with necrotic pulp, and sealing of perforations or apical surgeries, among others [1,2]. BECs are powder formulations based on calcium silicates, mainly Ca_3_SiO_5_ and Ca_2_SiO_4_, which react with water and harden to form hydrated phases of good sealing abilities [1,3,4], antibacterial properties, and bioactive inducement of periapical healing and hard tissue formation [5]. Irrespective of their variable composition, the bioactivity, defined as the potential to stimulate the natural remineralization process at the material–tooth interface, is a common property of endodontic cements [1]. The international standard ISO 22317 [6] defines bioactive materials as those that—when implanted in a living organism—form a thin layer rich in calcium and phosphorus on the surface. Likewise, when crystallization of hydroxyapatite occurs on the material surface in simulated body fluid (SBF), the same bioactivity can be expected in vivo. Originally, this hydraulic tri/dicalcium silicate-based product was described as “hydrophilic powder composed of calcia, silica and alumina oxides” and was named “mineral trioxide aggregate” (MTA), which is still a generic name commonly used for BECs in dentistry [4].

However, despite its excellent properties, there are some problems in its clinical application that require improvement. Among the main disadvantages of its use are long setting time, difficulty in handling in the clinical setting, and its aging or biodegradation behavior once applied, producing undesirable dental discolorations. In recent years, manufacturers have been trying to solve these problems with changes in the formulations [7]. Hence, there is much interest in determining which powder parameters, i.e., crystal phase composition [8], particle size [9], or the uses of additives [10], are the strongest contributors to cement setting performance and bioactivity. Particle size and specific surface area-related parameters have been shown to have an important effect on setting time and final consolidated microstructure. A larger surface area will increase powder reactivity towards hydration, promoting setting properties. In this respect, BEC formulations of high surface area have been synthesized using sol–gel procedures [11]. The sol–gel technique is a chemical procedure that consists in the successive hydrolysis and polycondensation of alkoxide precursors, typically tetraethoxysilane (TEOS), to develop colloidal particles (sol) and consequently convert them into a network (gel) of intrinsic nanoporosity [12], which confers to the material improved textural properties and functionalities [13,14]. A diversity of sol–gel materials in different configurations, i.e., powders, monoliths, foams, fibers, or coatings, are now under intensive investigation for biomedical applications [15,16,17,18].

Specifically in relation to endodontic cements, dicalcium and tricalcium silicates are the main components [3,19], and can be successfully improved in terms of textural properties by sol–gel synthesis [20,21]. However, the selection of the different synthesis variables, such as the type of reagents, their molar ratios, the sequence of incorporation and reaction times etc., is critical to control these material properties and requires further study. In this work, the sol–gel synthesis of a calcium silicate formulation with Ca/Si stoichiometry equal to 3 was studied, taking into account different preparation variables and how these affect the physicochemical properties of both the low-temperature sol–gel precursors and the final products after crystallization treatment. A first objective of the work was to optimize the synthesis variables in order to make the low-temperature sol–gel material amorphous, thus ensuring a reproducible and homogeneous precursor material as a step prior to the high-temperature treatment for the crystallization of the calcium silicate phases. Once a reproducible BEC material with an appropriate composition of silicate phases with hydraulic properties has been achieved, the next objective is to increase the textural parameters (i.e., specific surface area) of the BEC, and for this purpose we investigated an additional post-synthesis treatment using ethanol and its effect on the setting time and bioactivity results. To the authors’ knowledge, this post-synthesis treatment has not been previously published. The ethanol-treated BEC product is analyzed and compared with its untreated counterpart in terms of physicochemical properties, setting time, and in vitro bioactive response.

## 2. Materials and Methods

### 2.1. Bioactive Endodontic Cement (BEC) Synthesis Procedure

The chemical synthesis of the BEC powders is based on the sol–gel technique, which starts with the hydrolysis of the silicon alkoxide precursor in an acid medium. For this purpose, 200 µL of the acidic compound HNO_3_ (69.5%, Carlo Erba, Val de Reuil, France) or HCl (37%, Fluka, Austria) are added to 15 mL of DI water and left to stir for 5 min. Further, 8.48 mL of tetraethyl orthosilicate (TEOS; Si(OC_2_H_5_)_4_ 99%, Alfa-Aesar, Kandel, Germany) is added and stirred for 35 min. Then 26.57 g of the calcium precursor salt is added and stirred for another 70 min. For the calcium precursor salt, three commercial products were tested: Ca(NO_3_)_2_·4H_2_O (42353, Acros Organics, Mumbai, India), Ca(NO_3_)_2_·4H_2_O (C4955, Sigma-Aldrich, Mumbai, India), and CaCl_2_·2H_2_O (12312, Alfa Aesar, Karlsruhe, Germany). This is all done at room temperature. The reagent products and the nomenclature used for the samples are listed in Table 1. The solution obtained is transferred to a crystallizer (180 mm diameter) and placed for 24 h in an oven at 60 °C first and then for another 24 h at 120 °C. BEC powders are obtained by heat treatment at 1400 °C of the sol–gel precursors. For this purpose, a temperature ramp of 1 °C/min is used until 1400 °C is reached and it is maintained at this temperature for 2 h. The obtained materials are finally sieved using 32 µm mesh.

### 2.2. Ethanol Post-Synthesis Treatment (EPT)

In order to further reduce the particle size of the selected BEC material, additional grinding treatment and subsequent liquid-phase filtration using ethanol was implemented. For this, 3.5 g of BEC material was manually grinded and dispersed in 50 mL of ethanol (1.00983 Emsure, Merck Millipore, Darmstadt, Germany) for 15 min using an ultrasonic bath. The slurry was then filtered using a 10 µm sieve and the ethanol evaporated, first naturally at room temperature in a fume hood for 24 h and further in an oven for 2 h at 60 °C. Hereafter, BEC samples that underwent this treatment are identified as “EPT.”

### 2.3. Material Characterization

#### 2.3.1. X-ray Diffraction (XRD) Analysis

This technique was used as the main tool to evaluate the influence of sol–gel synthesis variables for the first objective of the work. Thus, the amorphous or crystalline nature of the low-temperature sol–gel precursors and the formation of the crystallization compounds and their different forms with subsequent heating treatment were determined. Further, it was used to determine the effect of post-synthesis treatment with ethanol on the crystalline phases of the non-hydrated material and after the hydration process, and to analyze the deposition of calcium phosphate phases in bioactivity tests. It was performed using a Panalytical X’Pert Pro diffractometer (Almelo, the Netherlands) and Cu(Copper)-Kα (0.154187 nm) radiation. The measurements were performed at 40 kV and 20 mA using a step size of 0.02 and exposure time of 500 s. The identification of the phases was carried out by the X’Pert HighScore Plus pattern analysis tool utilizing the international Center for Diffraction Data (ICDD) database (2002, Newtown Square, PA, USA).

#### 2.3.2. Particle Size Measurements

These were carried out with the non-hydrated material to determine the effect of EPT on particle size. They were conducted in a Malvern Sizer laser diffraction instrument (Southborough, MA, USA) on ultrasonic bath dispersions (15 min) prepared with 40 mg sample in 50 mL ethanol (1.00983 Emsure, Merck Millipore). Measurements were performed three times for each material group.

#### 2.3.3. Fourier-Transform Infrared (FT-IR) Spectroscopy

This technique has been used to evaluate the effect of EPT on the properties of non-hydrated material, as well as to assess the hydration process and the formation of calcium phosphate compounds after bioactivity tests. The spectra were collected in transmission configuration using 4 cm^−1^ intervals in a Nicolet IS50 FT-IR of Thermo Scientific (Madison, WI, USA).

#### 2.3.4. Field-Emission Gun Scanning Electron Microscopy (FEG-SEM)

Secondary electron observations were carried out to analyze the changes in the microstructure of the unhydrated and hydrated BEC material surfaces after EPT, as well as the formation of calcium phosphate compounds after bioactivity tests. These observations were complemented by chemical analysis using energy-dispersive X-ray analysis. A Hitachi S-4800 (Tokyo, Japan) microscope, at accelerating voltage of 2 kV, was used for this study. Energy-dispersive X-ray (EDX) analysis was performed at 20 kV with an EDX Bruker XFlash 4010 (Berlin, Germany) detector.

#### 2.3.5. Characterization of Textural Properties

Nitrogen physisorption measurements were carried out on a Micromeritics TriStar 3020 gas adsorption analyzer (Norcross, GA, USA) after degassing at 250 °C for 2 h in a nitrogen stream. The specific surface area was determined by the BET (Brunauer–Emmett–Teller) method, and the total pore volume was obtained from the N_2_ amount adsorbed at 0.99 relative pressure [22,23]. Measurements were performed three times for each material group.

#### 2.3.6. Hydration Protocol and Setting Time Analysis

The hydration properties and setting time were studied for the materials mixed in the ratio of 1 g of powdered material and 0.6 mL of Milli-Q water. The paste with homogeneous consistency was compacted in silicone molds of 10 mm in diameter and 4 mm high, and stored in an incubator at 37 °C and 95% relative humidity. The setting time was measured according to the American Society for Testing and Materials (ASTM) specification C 266, and 113.4 ± 0.5 g and 453.6 ± 0.5 g Gillmore needles were used, respectively, to determine the initial and the final setting times. This procedure was repeated at 60 s intervals, and the time was measured using a digital chronometer. The setting times were measured from the start of mixing to the time at which no indentations could be seen on the surface of the specimen. Measurements were performed three times for each material group.

#### 2.3.7. Bioactivity Assays

Bioactivity was evaluated by soaking the cement disks in simulated body fluid (SBF) during 7 days in an incubator at 36.5 °C and 60 rpm shaking [3,24]. Prior to the bioactivity assay, the samples were sterilized under UV light for 10 min on each side. SBF solution was filtered using a 0.2 mm bacteriostatic filter (Biofil).

## 3. Results

The results are organized in two sections according to the two objectives set out in the introduction. The first subsection shows the influence of sol–gel synthesis variables, such as: (i) the silicon reactive (TEOS) hydrolysis time, (ii) the mixing order of the two reagents TEOS and calcium salt, and (iii) the calcium salt and acid reactive sources, in relation to the product reproducibility and standardization of both the low temperature sol–gel precursor and the final calcium silicate phases and polymorphs after heating treatment. The second subsection shows the effect of a novel ethanol-based treatment, implemented after BEC synthesis (post-synthesis), on the physicochemical properties, setting time, and in vitro bioactivity of the most optimal hydraulic composition selected from the previous study.

### 3.1. Sol–Gel Synthesis Variable Influence in the BEC Powder Product Standardization and Optimization

The XRD results are presented below, including both the low-temperature precursor material and the high-temperature treated material, for the mentioned three types of variables that showed the most interesting effects.

**Figure 1:**
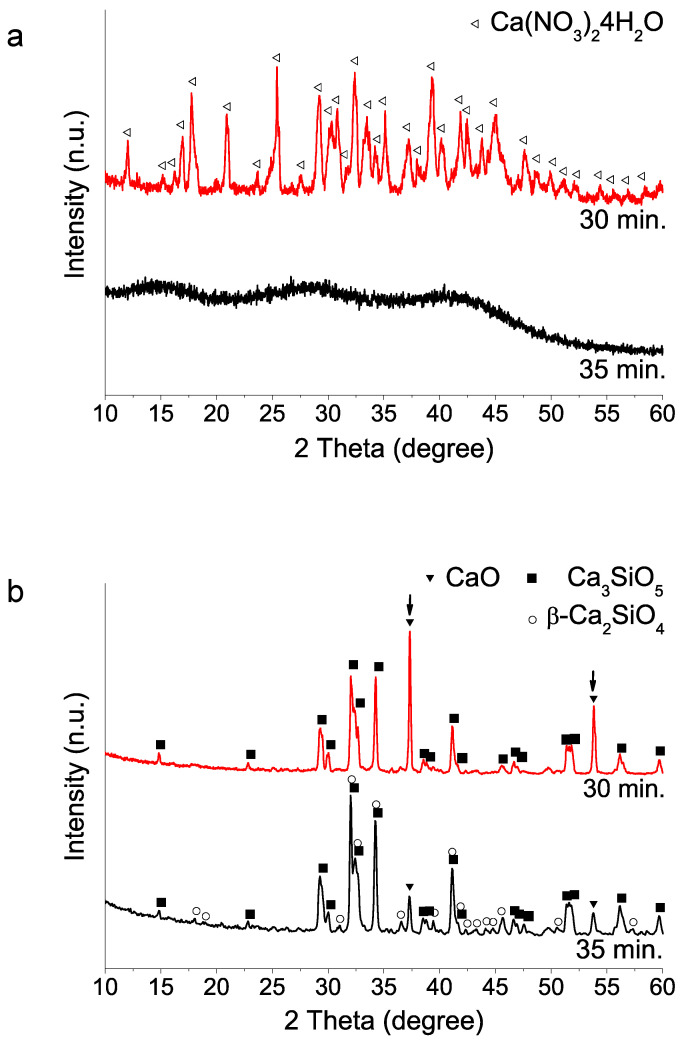
XRD patterns of sol–gel BEC preparations using different TEOS hydrolysis step times: (**a**) as synthesized powders (without heating treatment); (**b**) after heat treatment at 1400 °C.

**Figure 2:**
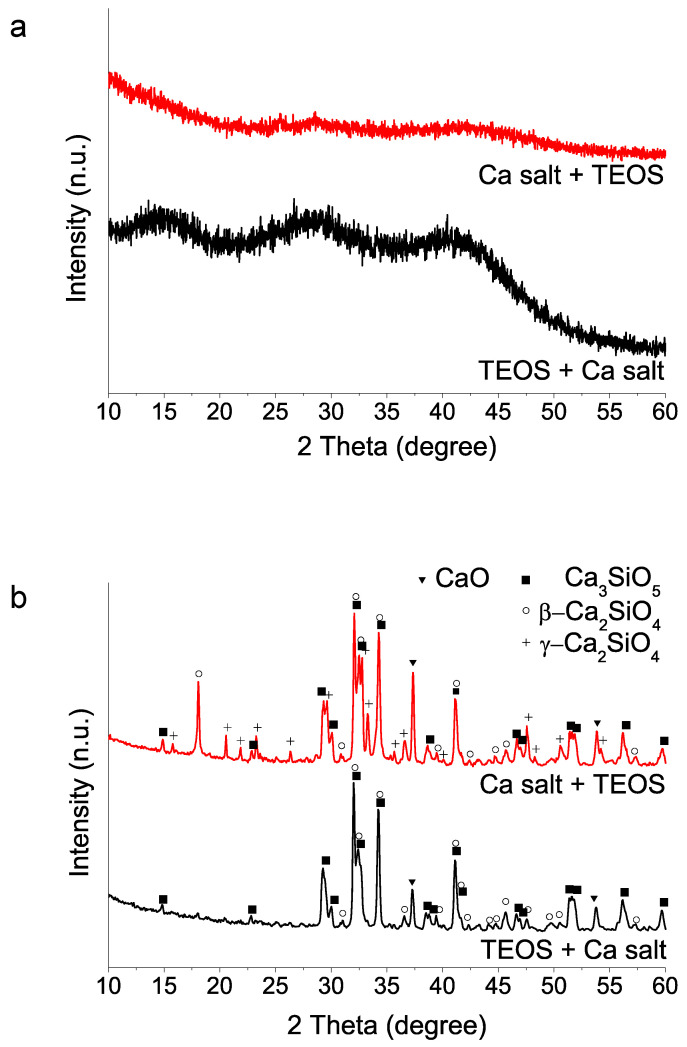
XRD patterns of sol–gel BEC preparations using different addition order of reagents: (**a**) as-synthesized powders (without heating treatment); (**b**) after heat treatment at 1400 °C.

**Figure 3:**
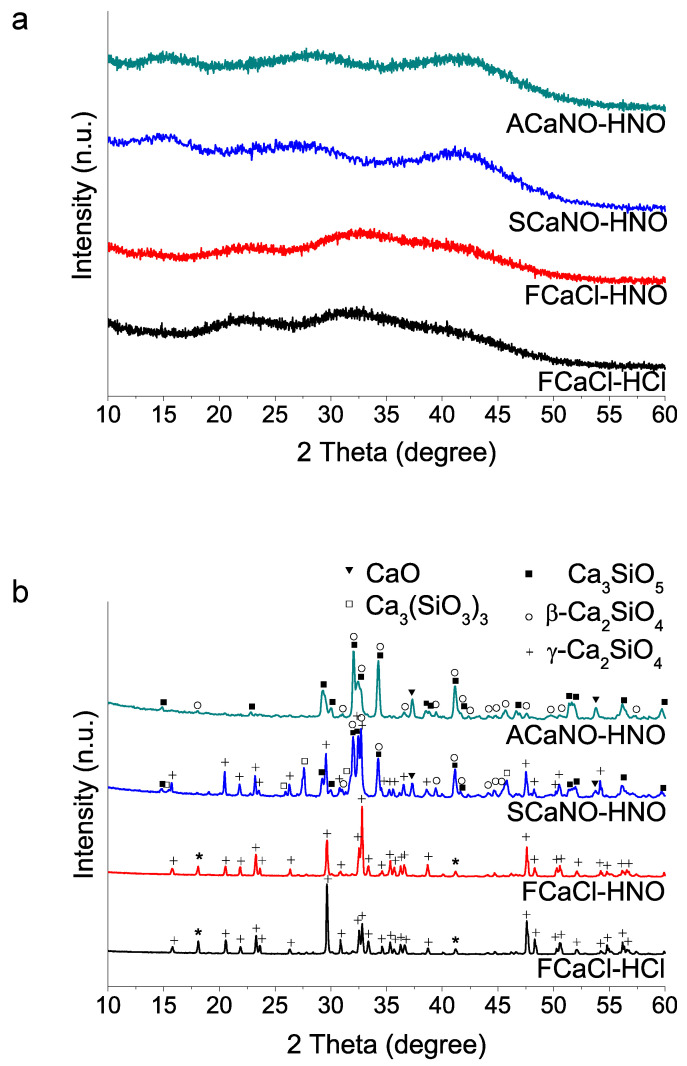
XRD patterns of sol–gel BEC preparations using different reactive sources as specified in Table 1: (**a**) as-synthesized powders (without heating treatment); (**b**) after heat treatment at 1400 °C. The two peaks marked with asterisks could not be matched to any phase of the database records.

(i) Hydrolysis reaction time of the silicon reagent (TEOS): Based on Lee et al.’s work [11] on the best formulation, we found that the TEOS hydrolysis time variable was a critical parameter to achieve an amorphous sol–gel precursor. Figure 1a illustrates how small time variation from 30 to 35 min of the nitric acid catalyzed TEOS hydrolysis step can significantly influence calcium nitrate salt mixing and dissolution, as it was confirmed by the XRD peaks detected for the synthesis performed using shorter times than 35 min. Further, the presence of undissolved Ca(NO_3_)_2_4H_2_O can influence ulterior 1400 °C HT crystal phase formation. Figure 1 shows how the precursor containing calcium nitrate crystals results in a final product with higher CaO content than obtained from its amorphous counterpart (Figure 1b).

(ii) Mixing order of the two reagents TEOS and calcium salt: The addition orders of TEOS and Ca(NO_3_)_2_ 4H_2_O reactants strongly influence 1400 °C HT crystallization process (Figure 2). The addition of TEOS first yields a higher proportion of tricalcium silicate after 1400 °C HT. In contrast, the addition of the calcium salt into the water–nitric acid mixture first seems to hinder the hydrolysis of TEOS, in view of the longer times needed for the mixture to acquire a homogeneous appearance. Besides, the subsequent HT at 1400 °C results in a product with a much higher proportion of dicalcium silicate phase, both as beta and gamma polymorphs.

(iii) Sources used in calcium salt and acid compound reagents: Using TEOS addition at first and selected 35 min acid catalyzed hydrolysis time, we then studied the calcium and acid reactive sources. Sample identification is listed in Table 1 above. The results (Figure 3) indicate that the use of the calcium nitrate salt, both Sigma-Aldrich and Acros Organics, in combination with HNO_3_ produce final products with high content of tricalcium silicate. Besides, formation of gamma dicalcium silicate polymorph is not detected using the Acros Organics salt. On the contrary, when calcium chloride is used, and for the case of the two acid reagents HCl and HNO_3_, HT at 1400 °C generates dicalcium silicate in its gamma form (Powder Diffraction File (PDF) 01-087-1256) as the only crystalline phase detected (Figure 3b).

### 3.2. Effect on the BEC Properties of the Ethanol-Based Post-Synthesis Treatment

Based on the results obtained in the previous section, the ACaNO-HNO formulation, showing the highest content of Ca_3_SiO_5_ as well as the absence of the non-hydraulic phase γ-Ca_2_SiO_4_, was selected to perform further ethanol post-synthesis treatment (EPT).

#### 3.2.1. Influence of EPT on the Physicochemical Properties of Unhydrated Sol–Gel BEC Material

**Figure 4:**
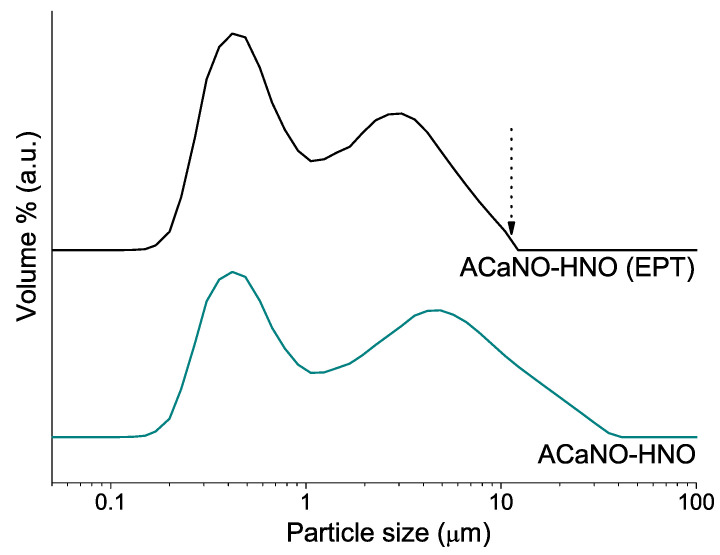
Particle size distributions of the sol–gel synthesized powders: without post-synthesis treatment (ACaNO-HNO), and after EPT (ACaNO-HNO (EPT)).

Figure 4 displays particle size distributions for the as-synthesized BEC and also after EPT. For both samples, the graph shows a bimodal distribution of two maxima at 0.3–0.4 and 5–7 μm ranges. As expected, all the particles above 10 μm are completely removed after the EPT.

**Figure 5:**
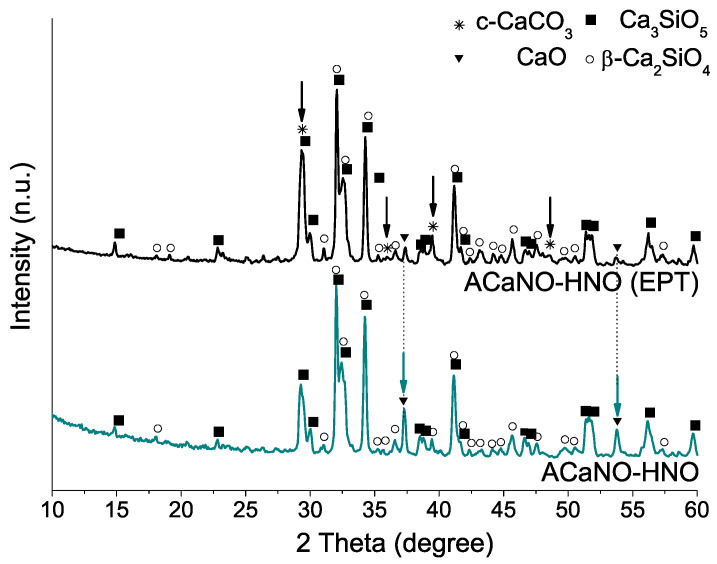
XRD patterns of unhydrated sol–gel BECs heat-treated at 1400 °C: without post-synthesis treatment (ACaNO-HNO), and after EPT (ACaNO-HNO (EPT)).

XRD analysis (Figure 5) indicates that EPT does not significantly affect formed calcium silicate phases, although it does result in the growth of new calcium carbonate crystals in the form of the calcite polymorph, being those at the expense of the calcium oxide phase, as suggested by its diffraction peaks decreasing intensity.

**Figure 6:**
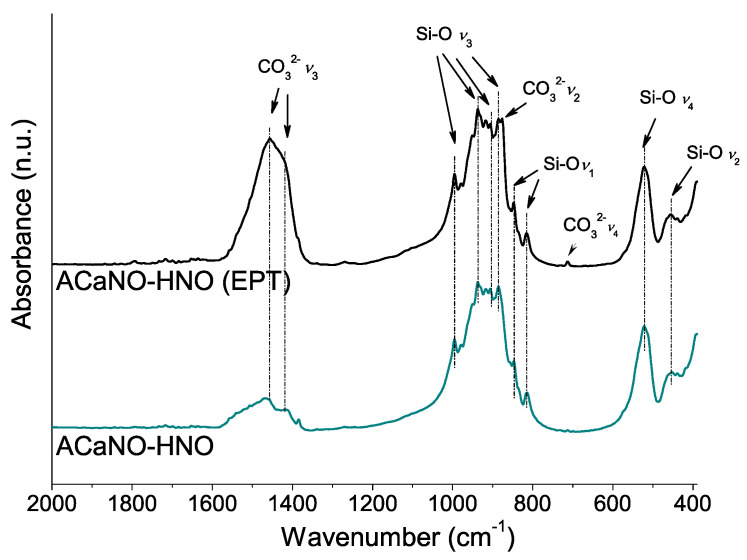
FT-IR analysis of unhydrated sol–gel BECs heat-treated at 1400 °C: without post-synthesis treatment (ACaNO-HNO), and after EPT (ACaNO-HNO (EPT)).

This growth of calcium carbonate caused by EPT is confirmed by FT-IR analysis (Figure 6) showing the appearance of additional bands corresponding to calcite-specific vibrational modes at 874 and 713 cm^−1^ [25,26]. Besides, vibrational bands corresponding to Si-O asymmetric stretching (*ν*_3_) at 994, 936, 906, and 886 cm^−1^, and Si-O bending *ν*_4_ and *ν*_2_ at 520 and 456 cm^−1^, respectively, characteristics of Ca_3_SiO_5_ [3] are displayed in Figure 6. Interestingly, the presence of the band at 814 cm^−1^ associated with the Si-O symmetric stretching (*ν*_1_) indicates the ideal [SiO_4_]^4−^ tetrahedron structure distortion of the Ca_3_SiO_5_ silicate phase [27]. This result suggests that Ca_3_SiO_5_ is in the form of a triclinic structure polymorph in good agreement with the XRD analysis (PDF 00-031-0301). The Si-O band at 846 cm^−1^ has been assigned to dicalcium silicate (belite) [28].

**Figure 7:**
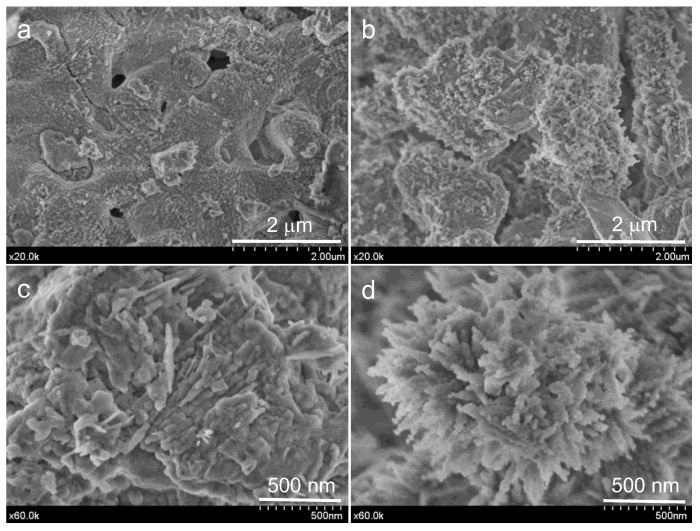
FEG-SEM observations at different magnifications of unhydrated ACaNO-HNO formulation: (**a**,**c**) secondary electron (SE) images of the material without post-synthesis treatment; and (**b**,**d**) SE images of the material after EPT.

**Figure 8:**
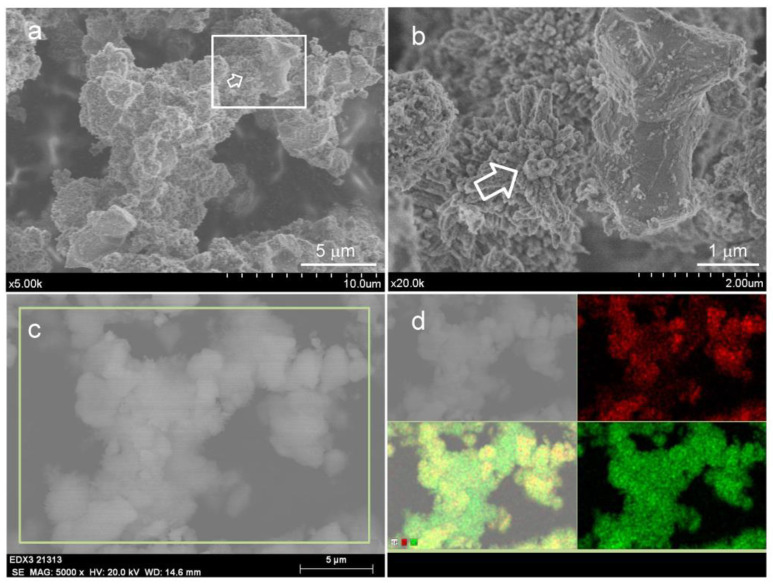
FEG-SEM observations and EDX analysis of ACaNO-HNO (EPT): (**a**) low-magnification secondary electron (SE) image using 2 kV; (**b**) higher magnification corresponding to the area marked with the white square in image (**a**), white arrow in images (**a**,**b**) points to a characteristic aggregate formed by the EPT treatment; (**c**) low-magnification SE image of the area displayed in (**a**) using 20 kV; (**d**) EDX analysis of image (**c**) showing Si (red), Ca (green), and merged (yellow) element-distribution analysis.

Figure 7 displays FEG-SEM images at different magnifications of ACaNO-HNO formulation, comparatively for the as-synthesized and after EPT. The b, d, and f micrographs, corresponding to the EPT material, show a less flat surface that appears decorated by aggregates with rhombic morphology characteristic of calcium carbonate crystals in the calcite polymorph [25]. An EDX analysis of these aggregates is displayed in Figure 8, indicating that these morphologies correspond to very rich calcium areas, which are at the same time deficient in silicon, thus confirming the calcite growth after EPT.

Physisorption measurements can help to quantify the structural and morphological changes qualitatively observed above. In fact, the results indicate that the EPT produces a threefold increase in the values of the specific surface area and pore volume (nanopore) parameters (Table 2).

#### 3.2.2. Influence of EPT on Hydration and the Setting Properties of the Sol–Gel Synthesized BEC Material

**Figure 9:**
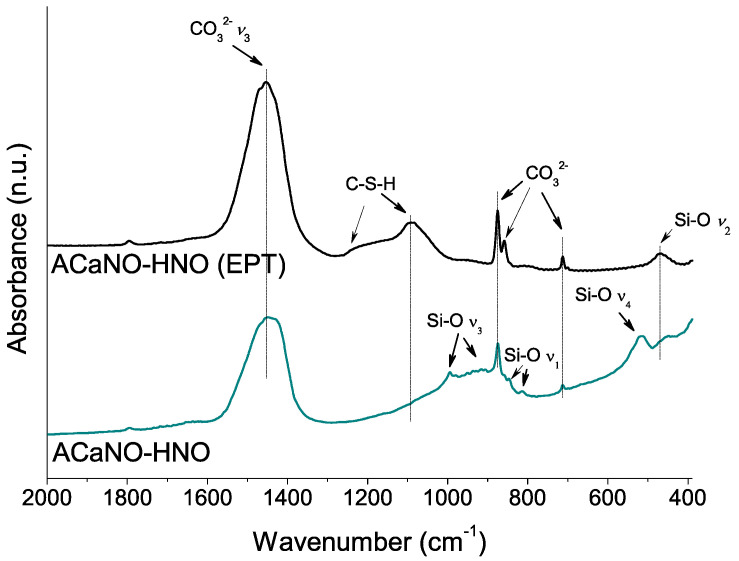
FT-IR analysis of hydrated BECs: without post-synthesis treatment (ACaNO-HNO), and after EPT (ACaNO-HNO (EPT)).

The hydration process was analyzed using FT-IR (Figure 9). Both type samples show significant increase of carbonate bands at 1450, 875, 855, and 714 cm^−1^ built up from the reaction of atmospheric CO_2_ with calcium ad species. However, the distinctively ACaNO-HNO (EPT) sample shows stronger shifting of the Si-O asymmetric stretching (*ν*_3_) from ~950–1000 cm^−1^ to higher wave numbers ~1100 cm^−1^ as well as changes in the relative intensities of the out of plane (*ν*_4_) and in plane (*ν*_2_) bending vibrations [27], which are indicative of calcium silicate unit (SiO_4_^4−^) dissolution and polymerization to form CSH [3].

**Figure 10:**
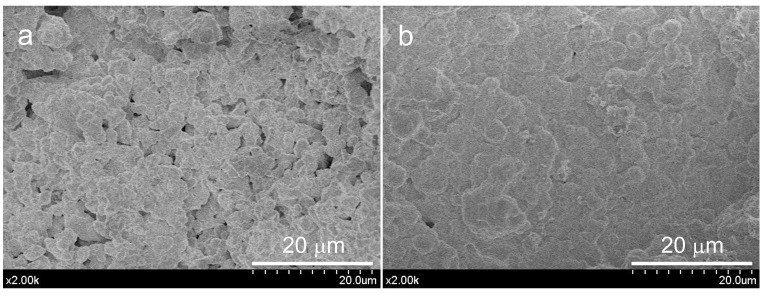
FEG-SEM observations on fracture surfaces of the hydrated ACaNO-HNO formulation: (**a**) without post-synthesis treatment; and (**b**) implementing EPT.

The comparison of fracture surface FEG-SEM observations of studied hydrated materials indicates that the alcohol-processed sample shows lower porosity between the precursor microparticles, appearing as a more sealed surface (Figure 10).

The effect of the EPT was functionally evaluated by determining the initial and final setting times as displayed in Table 3. The results indicate a very clear effect on the reduction of the setting time of the BEC material after EPT.

#### 3.2.3. Influence of EPT on the Bioactivity of the Sol–Gel Synthesized BEC Material

**Figure 11:**
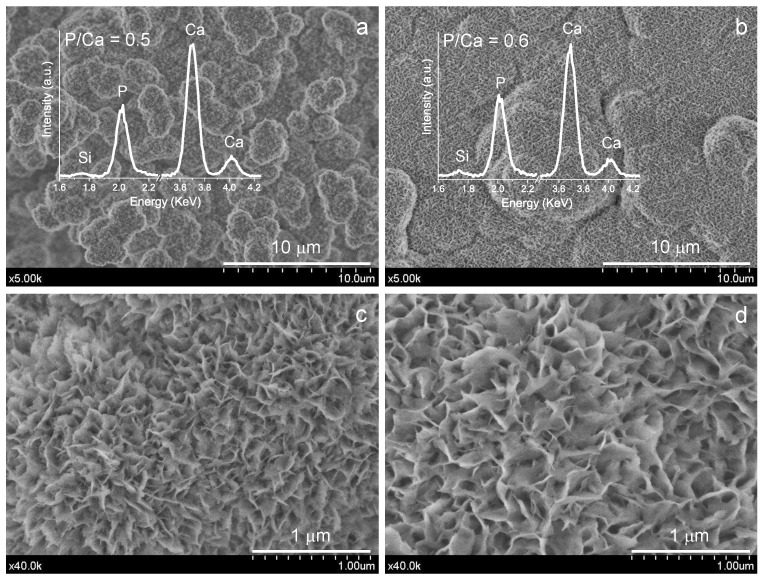
FEG-SEM observations and EDX analysis of hydrated ACaNO-HNO formulation surfaces after 7 days SBF essay: (**a**,**c**) without post-synthesis treatment; and (**b**,**d**) after EPT.

The surface microstructure observations and analysis of BEC samples after 7 days’ SBF soaking using FEG-SEM are displayed in Figure 11. Typical calcium phosphate (CaP) aggregates of spherical shape showing average diameters in the 1–2 μm range are observed for the untreated sample. Comparatively, for the EPT sample, the observed growth in size of these spherical aggregates seems to culminate in their coalescence, and the lamellar formations of CaP appear more visible due to their larger size.

**Figure 12:**
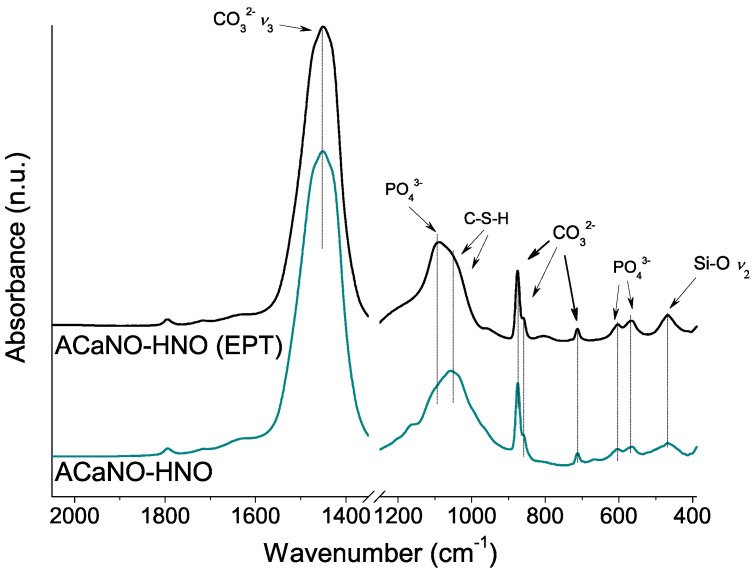
FT-IR analysis of hydrated BEC surface after 7 days SBF essay: without post-synthesis treatment (ACaNO-HNO), and after EPT (ACaNO-HNO (EPT)).

To further characterize the CaP layer formed for both samples, FT-IR and XRD analyses were performed using material obtained by surface scraping. In comparison with the FT-IR analysis of the hydrated samples before the SBF essay (above, in Figure 6), both samples show an intensity increase of the carbonate bands at 1450, 875, 855, and 714 cm^−1^ as well as the calcium silica hydrate CSH band within the 1000–1100 cm^−1^ range. New specific phosphate phase bands at 1097, 960, 607, and 570 cm^−1^ are now detected in both samples (Figure 12).

**Figure 13:**
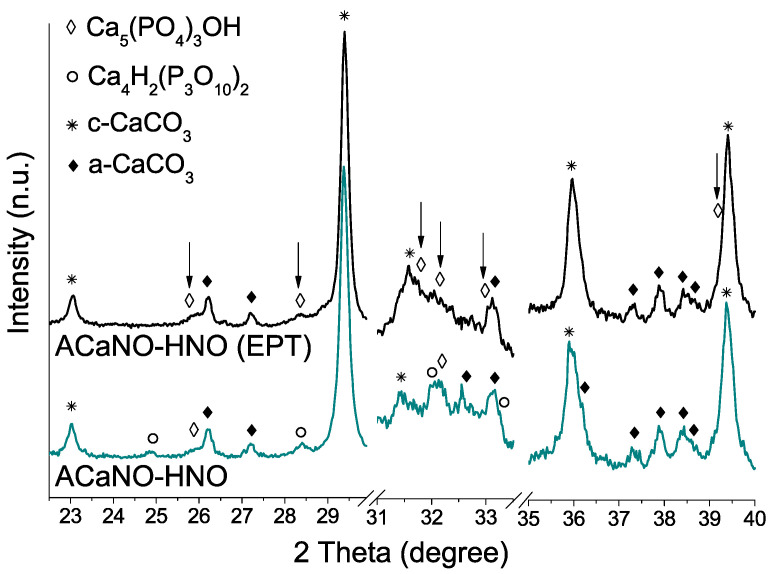
XRD analysis of hydrated BEC surface after 7 days SBF essay: without post-synthesis treatment (ACaNO-HNO), and after EPT (ACaNO-HNO (EPT)).

As regards de CaP crystalline phase formation (Figure 13), the most intense XRD peaks for hydroxyapatite phase (PDF 00-009-0432) at 2θ = 31.7, 32,2 and 32.9 are clearly detected for the EPT sample, whilst for the untreated sample, main signal matching with calcium hydrogen phosphate (PDF 00-017-0934) at 2θ = 24.8, 28.4, 32.1, and 33.3 are prevalent. Calcium carbonate in the form of calcite (PDF 01-086-2334) and aragonite (PDF 00-041-1475) are predominant.

## 4. Discussion

The sol–gel synthesis process is a versatile and low-cost route to produce new BEC materials of highly controlled homogeneity and chemical composition, as well as improved textural properties in terms of surface area and nanoporosity [11,29]. However, there is still not much work on their systematic use in the preparation of BECs and thus on the selection and influence of the most relevant synthesis parameters. The process requires a final temperature treatment step to produce the crystallization of the calcium silicate phases responsible for the hydraulic properties of these materials. In this respect, diffraction analysis of the sol–gel precursor obtained at low temperature is not usually specified in the literature, and we believe that a very good measure to guarantee the reproducibility of phase crystallization as well as the final product homogeneity is to ensure the use of amorphous precursors. In this report, care has also been taken to characterize this low-temperature precursor. The large number of process variables involved, such as the type and brand of the precursor compounds, their incorporation protocol, reaction times, and temperatures of the synthesis and subsequent treatments, all have an important influence on the final product.

In this work, we used a Ca/Si reactive molar ratio of 3 (the same stoichiometry as Ca_3_SiO_5_), and studied variations on successful procedures accepted in the literature [11,21]. We found that a time of less than 35 min in the first stage of TEOS hydrolysis can hinder the dissolution of the calcium nitrate precursor salt, and this in turn is ultimately reflected in a higher CaO ratio after HT. The addition orders of TEOS and Ca(NO_3_)_2_ 4H_2_O reactants also strongly influence 1400 °C HT crystallization. Particularly relevant is the influence of the type of precursor calcium salt used, nitrate or chloride, on the proportion of tri(C3S)/di(C2S)calcium silicate products obtained. Although both salts bring about sol–gel glass-polymer precursors, the use of calcium nitrate promotes the subsequent crystallization of tricalcium silicate (C3S). Calcium chloride salt instead, either using HNO_3_ or HCl as catalyst generates the formation of dicalcium silicate (C2S) in its gamma form, this being the only phase detected. On the other hand, the nitrate Acros reagent yields Ca_3_SiO_5_ (C3S) as major product, with additional weak peaks of β-Ca_2_SiO_4_ (β-C2S) phase being detected, whilst the Sigma-Aldrich reagent produces much higher proportion of C2S phase, particularly in the gamma polymorph (γ-C2S). Although C3S and C2S are the two principal components of hydraulic cements, C3S shows much faster hydration kinetics [30,31], and has been proclaimed to be mainly responsible for strength development in the cement [8]. In this respect, C2S and its polymorphism has been the subject of many studies in relation to slow hydration activity [31,32]. Particularly, the gamma (γ-C2S) phase has been reported to not hydrate and then, the more reactive β polymorph needs to be stabilized in cement powders. This has been achieved by the presence of reactant impurities [33] or the incorporation of stabilizing additives, such as N_2_O, K_2_O, BaSO_4_, and Al_2_O_3_ [34,35]. Our results suggest that using calcium nitrate reagents from two different brands results in significant differences in the ratio of the C2S/C3S phases and in the stabilization of polymorphs of the C2S phase after the HT. Thus, the Sigma salt produces more C2S in the gamma form, a phase that is not detected using the Acros salt. A detailed look at the certificates of analysis of both salts indicates that there are some differences in the ion trace content (Table A1, Appendix A). In particular, the Acros salt specifies a somewhat higher content of Na, Ba, and Sr.

Another finding from our XRD and FT-IR analysis is that C3S identified is present in its triclinic structure is in good agreement with previous work by Lee et al. [11] which, also identifies C3S in this form, for a material prepared by the sol–gel method. In contrast, studies of commercial MTA-type materials obtained by conventional methods, such as ProRoot MTA [3,36] and MTA Angelus [37], report monoclinic C3S. C3S exhibits polymorphism in the triclinic, monoclinic, and rhombohedral forms depending on the temperature and impurities [38]. It has been reported that the structural differences between the polymorphs include slight shifts of the atomic positions and changes in the orientations of the [SiO_4_]^4−^ tetrahedra, which show varying degrees of disorder [27,38]. Whilst the atomic structure of the different polymorphs and stability is still not fully understood, its influence in hydration reactivity is another important aspect that merits further research [30,39].

Once the formulation with the highest C3S content, ACaNO-HNO, has been selected, the use of an alcohol-based post-synthesis treatment is investigated to reduce the particle size and then to increase the specific surface area and the potential increase in the BEC reactivity upon hydration to form calcium hydroxide and calcium silicate hydrate (CSH). Physisorption data confirm the increase of more than 200% in its specific surface area with this treatment. This observed effect on textural properties is also accompanied by an increase in the detection by XRD and FT-IR of carbonate species in the form of calcite. The FEG-SEM observations and EDX mapping analysis confirm also that EPT leads to the growth of aggregates with elongated grain morphologies characteristic of calcite, which may well contribute to an additional increase in the surface area. Interestingly, Biodentine, another hydraulic cement widely used in the clinic for its low setting time of 10–12 min [40], which is marketed in two components, powder and liquid, has some characteristics in common with our EPT material. The powder is composed of 80% C3S in its triclinic form, also contains a high 15% content of CaCO_3_, and has low particle size [9,37,40]. However, it should also be noted that the liquid contains calcium chloride, which acts as a setting accelerator [37].

The long setting time is one of the shortcomings limiting the clinical application of BECs. In this respect, our selected sol–gel material ACaNO-HNO shows much lower values than those observed for commercial products, such as ProRoot White MTA, NeoMTA Plus, or HP Repair [3]. Among the different variables that can influence the setting time, such as the particle size [9] or the ratio of the different crystalline phases [8,10], one main difference of the presented material ACaNO-HNO with respect to the commercial BECs is the nanoporosity, derived from the synthesis process and resulting in a higher specific surface area for the hydration reaction [11]. However, this paper’s most novel observation is the large additional reduction in setting time observed after EPT. The characterization data obtained indicate that EPT produces a very significant increase in specific surface area and the growth of surface carbonate aggregates in the form of calcite. XRD analysis suggests that calcium carbonate is formed by reaction of CaO and atmospheric CO_2_ during treatment and in good agreement, while the FT-IR spectra show no changes in the bands of the calcium silicates. The carbonation of cements has attracted extensive interest as an effective approach to sequestrate CO_2_ as well as to improve the properties of cement-based materials at the same time [41,42,43]. Studies indicate that CaCO_3_ particles act as crystallization sites for the newly formed hydrates, accelerating the hydration process [42]. In fact, there is an endodontic cement on the market, MicroMega-MTA, with a reduced setting time of 20 min, and the manufacturer claims this reduction due to the addition of CaCO_3_ [44]. Ca(OH)_2_ but also C-S-H and unhydrated cement components C3S and C2S can consume CO_2_ and get involved in the carbonation process [45]. It is reported that carbonation leads to reduction in porosity and consolidate the microstructure of the hardened cement paste resulting in an increase of strength [45,46]. Besides, this ability of the material to absorb CO_2_, which can be stored in the periradicular tissue of the root canal cavity, adds another interesting aspect for clinical use by eliminating the atmosphere essential for the survival of anaerobic bacteria [47].

Bioactivity, on the other hand, is an important property of endodontic cements [1]. Defined in terms of the in vitro evaluation for apatite-forming ability of implant materials [6], has been shown that effectively cloaks the foreign body from the tissue and allows wound healing [4]. Particularly, in vitro evaluation of hydroxyapatite-forming ability on the BEC surface in SBF is useful for evaluating its potential to stimulate the natural remineralization process at the material–tooth interface [24] and the regeneration of periradicular tissue [1]. The superficial formation of calcium phosphate thus promotes wound healing and formation of a reparative dentinal barrier whilst upregulating the differentiation of osteoblast, fibroblast, cementoblast, odontoblast, pulp cells, and many stem cells [48]. In odontoblast and cementoblast, high pH-sensing mechanisms are important for reactivating mineralization induced by an alkaline environment [49]. In this respect, our measurements of the SBF solution 7 days into the experiment indicate a significant increase in pH, which increases to values of 8.0 and 8.4 for the newly synthesized material and the EPT, respectively. Endodontic studies of the use of calcium hydroxide and its resulting alkaline environment created in the proximity of or adjacent to this material have been related to the influence of various mineralization enzymatic pathways as well as an antibacterial pH gradient [50]. Our results indicate that EPT stimulates more the bioactive response of the material, leading to increased growth and crystallization of the surface calcium phosphate layer. Both the volumetric expansion of the BECs and their ability to trigger the nucleation and growth of apatite deposits may reduce porosity and ensure a long-lasting seal. Furthermore, the strong presence of calcite crystals could have an additional advantage of the presented sol–gel BEC materials to attract fibronectin, which is responsible for cellular adhesion and differentiation [51,52].

## 5. Conclusions

The sol–gel synthesis technique is effective for obtaining BECs with majority C3S content in the triclinic form. The control of the set of synthesis variables tested allows obtaining a final material with an optimum formation of C3S and C2S in its beta form, thus avoiding the crystallization of C2S in its non-hydraulic gamma polymorph.

The implementation of a novel post-synthesis treatment at room temperature using ethanol allows for a final BEC product with a finer particle size and increased CaCO_3_ content, resulting in an improved material in terms of setting time and bioactive response.

## Figures and Tables

**Table 1:** Synthesis reagents and nomenclature used for the different preparations.

Sample Name	Ca Salt Reactive	Acid-Type Reactive
ACaNO-HNO	Ca(NO_3_)_2_·4H_2_O Acros Organics	HNO_3_ Carlo Erba
SCaNO-HNO	Ca(NO_3_)_2_·4H_2_O Sigma Aldrich	HNO_3_ Carlo Erba
FCaCl-HNO	CaCl_2_·2H_2_O Alfa Aesar	HNO_3_ Carlo Erba
FCaCl-HCl	CaCl_2_·2H_2_O Alfa Aesar	HCl Fluka

**Table 2:** BEC samples’ textural parameters obtained by N_2_ physisorption.

Sample	S_BET_ (m^2^ g^−1^)	V_t_ (cm^3^ g^−1^)
ACaNO-HNO	2.6 ± 0.3	0.006 ± 0.000
ACaNO-HNO (EPT)	6.9 ± 0.2	0.021 ± 0.001

**Table 3:** Initial and final setting times measured for the sol–gel BEC samples.

Sample	Initial Setting (min)	Final Setting (min)
ACaNO-HNO	26 ± 1	95 ± 7
ACaNO-HNO (EPT)	6 ± 0	53 ± 4

## Appendix A

**Table A1:** Calcium nitrate salt reagent specifications ^1^.

Result Lot Numbers	C4955 Sigma Aldrich MKBQ0375V	42,353 Acros Organics A0413021
Chloride (Cl)	≤0.005%	≤0.005%
Sulfate (SO_4_)	≤0.002%	≤0.002%
Magnesium (Mg)	≤0.01%	0.0068
Potassium (K)	≤0.001%	0.0006%
Sodium (Na)	≤0.001%	0.0012%
Iron (Fe)	≤0.0001%	≤0.0005%
Aluminum (Al)	≤0.001%	-
Phosphorous (P)	≤0.0002%	-
Strontium (Sr)	-	0.0017%
Barium (Ba)	-	≤0.005%

^1^ The full certificate of analysis can be downloaded at https://www.acros.com/portal/alias__Rainbow/lang__en/tabID__43/DesktopDefault.aspx and https://www.sigmaaldrich.com/ES/en/search (accessed on 24 September 2021).

## References

[1] Parirokh M., Torabinejad M., Dummer P.M.H. (2018). Mineral trioxide aggregate and other bioactive endodontic cements: An updated overview—Part I: Vital pulp therapy. Int. Endod. J..

[2] Torabinejad M., Parirokh M., Dummer P.M.H. (2018). Mineral trioxide aggregate and other bioactive endodontic cements: An updated overview—Part II: Other clinical applications and complications. Int. Endod. J..

[3] Jiménez-Sánchez M.C., Segura-Egea J.J., Díaz-Cuenca A. (2019). Higher hydration performance and bioactive response of the new endodontic bioactive cement MTA HP repair compared with ProRoot MTA white and NeoMTA Plus. J. Biomed. Mater. Res. Part B Appl. Biomater..

[4] Primus C.M., Tay F.R., Niu L.-N. (2019). Bioactive tri/dicalcium silicate cements for treatment of pulpal and periapical tissues. Acta Biomat..

[5] Donnermeyer D., Bürklein S., Dammaschke T., Schäfer E. (2019). Endodontic sealers based on calcium silicates: A systematic review. Odontology.

[6] (2014). Implants for Surgery—In Vitro Evaluation for Apatite-Forming Ability of Implant Materials.

[7] Hosoya N., Takigawa T., Horie T., Maeda H., Yamamoto Y., Momoi Y., Yamamoto K., Okiji T. (2019). A review of the literature on the efficacy of mineral trioxide aggregate in conservative dentistry. Dent. Mater. J..

[8] Liu W., Chang J., Yue Z. (2011). Physicochemical properties and biocompatibility of tricalcium and dicalcium silicate composite cements after hydration. Int. J. Appl. Ceram. Technol..

[9] Ha W.N., Bentz D.P., Kahler B., Walsh L.J. (2015). D90: The strongest contributor to setting time in mineral trioxide aggregate and Portland cement. J. Endod..

[10] Stephan D., Maleki H., Knöfel D., Eber B., Härdtl R. (1999). Influence of Cr, Ni, and Zn on the properties of pure clinker phases Part I. C3S. Cem. Concr. Res..

[11] Lee B.-S., Lin H.-P., Chan J.C.-C., Wang W.-C., Hung P.-H., Tsai Y.-H., Lee Y.-L. (2018). A novel sol-gel-derived calcium silicate cement with short setting time for application in endodontic repair of perforations. Int. J. Nanomed..

[12] Hench L.L., West J.K. (1990). The sol-gel process. Chem. Rev..

[13] Ramiro-Gutiérrez M.L., Santos-Ruiz L., Borrego-González S., Becerra J., Díaz-Cuenca A. (2016). In vitro stimulation of MC3T3-E1 sells and sustained drug delivery by a hierarchical nanostructured SiO_2_-CaO-P_2_O_5_ scaffold. Microporous Mesoporous Mater..

[14] Romero-Sánchez L.B., Borrego-González S., Díaz-Cuenca A. (2017). High surface area biopolymeric-ceramic scaffolds for hard tissue engineering. Biomed. Phys. Eng. Express.

[15] Díaz A., López T., Manjarrez J., Basaldella E., Martínez-Blanes J.M., Odriozola J.A. (2006). Growth of hydroxyapatite in a biocompatible mesoporous ordered silica. Acta Biomat..

[16] Owens G.J., Singh R.K., Foroutan F., Alqaysi M., Han C.-M., Mahapatra C., Kim H.-W., Knowles J.C. (2016). Sol-Gel materials for biomedical applications. Prog. Mater. Sci..

[17] Borrego-González S., Romero-Sánchez L.B., Blázquez J., Díaz-Cuenca A. (2018). Nanostructured hybrid device mimicking bone extracellular matrix as local and sustained antibiotic delivery system. Microporous Mesoporous Mater..

[18] Valdés-Sánchez L., Borrego-González S., Montero-Sánchez A., Massalini S., de la Cerda B., Díaz-Cuenca A., Díaz-Corrales F.J. (2022). Mesoporous silica-based nanoparticles as non-viral gene delivery platform for treating retinitis pigmentosa. J. Clin. Med..

[19] Jiménez-Sánchez M.C., Segura-Egea J.J., Díaz-Cuenca A. (2020). A microstructure insight of MTA Repair HP of rapid setting capacity and bioactive response. Materials.

[20] Gou Z., Chang J. (2004). Synthesis and in vitro bioactivity of dicalcium silicate powders. J. Eur. Ceram. Soc..

[21] Zhao W., Chang J. (2004). Sol-Gel synthesis and in vitro bioactivity of tricalcium silicate powders. Mater. Lett..

[22] Sing K.S.W., Everett D.H., Haul R.A.W., Moscou L., Pierotti R.A. (1985). Reporting physisorption data for gas/solid systems with special reference to the determination of surface area and porosity. Pure Appl. Chem..

[23] Jiménez-Sánchez M.C., Segura-Egea J.J., Díaz-Cuenca A. (2019). Physicochemical parameters—Hydration performance relationship of the new endodontic cement MTA Repair HP. J. Clin. Exp. Dent..

[24] Jiménez-Sánchez M.C., Segura-Egea J.J., Díaz-Cuenca A. (2019). MTA HP Repair stimulates in vitro an homogeneous calcium phosphate phase coating deposition. J. Clin. Exp. Dent..

[25] Ni M., Ratner B.D. (2008). Differentiation of calcium carbonate polymorphs by surface analysis techniques—An XPS and TOF-SIMS study. Surf. Interface Anal..

[26] Ylmén R., Jäglid U., Steenari B.-M., Panas I. (2009). Early hydration and setting of Portland cement monitored by IR, SEM and Vicat techniques. Cem. Concr. Res..

[27] Ren X., Zhang W., Ye J. (2017). FTIR study on the polymorphic structure of tricalcium silicate. Cem. Concr. Res..

[28] Taddei P., Modena E., Tinti A., Siboni F., Prati C., Gandolfi M.G. (2011). Vibrational investigation of calcium silicate cements for endodontics in simulated body fluids. J. Mol. Struct..

[29] Chen C.-C., Ho C.-C., Chen C.-H.D., Ding S.-J. (2009). Physicochemical properties of calcium silicate cements for endodontic treatment. J. Endod..

[30] Durgun E., Manzano H., Pellenq R.J.M., Grossman J.C. (2012). Understanding and controlling the reactivity of the calcium silicate phases from first principles. Chem. Mater..

[31] Hong S.-H., Young J.F. (1999). Hydration kinetics and phase stability od dicalcium silicate synthesized by the Pechini process. J. Am. Ceram. Soc..

[32] Nettleship I., Slavick K.G., Kim Y.J., Kriven W.M. (1992). Phase transformations in dicalcium silicate: I, fabrication and phase stability of fine-grained β phase. J. Am. Ceram. Soc..

[33] Groves G.W. (1983). Phase transformations in dicalcium silicate. J. Mater. Sci..

[34] Smith D.K., Majumdar A.J., Ordway F. (1961). Re-Examination of the polymorphism of dicalcium silicate. J. Am. Ceram. Soc..

[35] Pritts I.M., Daugherty K.E. (1976). The effect of stabilizing agents on the hydratation rate of β-C_2_S. Cem. Concr. Res..

[36] Belío-Reyes I.A., Bucio L., Cruz-Chavez E. (2009). Phase composition of ProRoot mineral trioxide aggregate by X-ray powder diffraction. J. Endod..

[37] Camilleri J., Sorrentino F., Damidot D. (2013). Investigation of the hydration and bioactivity of radiopacified tricalcium silicate cement, Biodentine and MTA Angelus. Dent. Mater..

[38] Dunstetter F., De Noirfontaine M.-N., Courtial M. (2006). Polymorphism of tricalcium silicate, the major compound of Portland cement clinker: 1. Structural data: Review and unified analysis. Cem. Concr. Res..

[39] Pustovgar E., Sangodkar R.P., Andreev A.S., Palacios M., Chmelka B.F., Flatt R.J., D’Espinose De Lacaillerie J.-B. (2016). Understanding silicate hydration from quatitative analyses of hydrating tricalcium silicates. Nat. Commun..

[40] Domingos Pires M., Cordeiro J., Vasconcelos I., Alves M., Quaresma S.A., Ginjeira A., Camilleri J. (2021). Effect of different manipulations on the physical, chemical and microstructural characteristics of Biodentine. Dent. Mater..

[41] Zajac M., Lechevallier A., Durdzinski P., Bullerjahn F., Skibsted J., Ben Haha M. (2020). CO_2_ mineralisation of Portland cement: Towards understanding the mechanisms of enforced carbonation. J. CO_2_ Util..

[42] Parvan M.-G., Voicu G., Badanoiu A.-I., Nicoara A.-I., Vasile E. (2021). CO_2_ sequestration in the production of Portland cement mortars with calcium carbonate additions. Nanomaterials.

[43] Chen T., Xu P., Gao X., Wang T., Qin L. (2022). New sights in early carbonation of calcium silicates: Performance, mechanism and nanostructure. Constr. Build. Mater..

[44] MicroMega. https://micro-mega.com.

[45] Liu X., Lin W., Chen B., Zhang F., Zhao P., Parsons A., Rau C., Robinson I. (2018). Coherent diffraction study of calcite crystallisation during the hydration of tricalcium silicate. Mater. Des..

[46] Morandeau A., Thiéry M., Dangla P. (2014). Investigation of the carbonation mechanism of CH and C-S-H in terms of kinetics, microstructure changes and moisture properties. Cem. Concr. Res..

[47] Primathena I., Nurdin D., Hermawan H., Cahyanto A. (2021). Synthesis, characterization, and antibacterial evaluation of a cost-effective endodontic sealer based on tricalcium silicate-white Portland cement. Materials.

[48] Prati C., Gandolfi M.G. (2015). Calcium silicate bioactive cements: Biological perspectives and clinical applications. Dent. Mater..

[49] Muramatsu T., Kashiwagi S., Ishizuka H., Matsuura Y., Furusawa M., Kimura M., Shibukawa Y. (2019). Alkaline extracellular conditions promote the proliferation and mineralization of a human cementoblast cell line. Int. Endod. J..

[50] Do Nascimento C., Issa J.P.M., Iyomasa M.M., Regalo S.C.H., Siéssere S., Pitol D.L., Wolga N.d.O., Pedrazzi V. (2008). Bone repair using mineral trioxide aggregate combined to a material carrier, associated or not with calcium hydroxide in bone defects. Micron.

[51] Seux D., Couble M.L., Hartmann D.J., Gauthier J.P., Maglore H. (1991). Odontoblast-like cytodifferentiation of human dental pulp cells in vitro in the presence of a calcium hydroxide-containing cement. Archs. Oral Biol..

[52] Faraco I.M., Holland R. (2001). Response of the pulp of dogs to capping with mineral trioxide aggregate or a calcium hydroxide cement. Dent. Traumatol..

